# Novel Peptides with Dual Properties for Treating *Pseudomonas aeruginosa* Keratitis: Antibacterial and Corneal Wound Healing

**DOI:** 10.3390/biom13071028

**Published:** 2023-06-23

**Authors:** Floriana Cappiello, Sudhir Verma, Xiao Lin, Isabel Y. Moreno, Bruno Casciaro, Debarun Dutta, Alison M. McDermott, Mark Willcox, Vivien J. Coulson-Thomas, Maria Luisa Mangoni

**Affiliations:** 1 Laboratory Affiliated to Pasteur Italia-Fondazione Cenci Bolognetti, Department of Biochemical Sciences, Sapienza University of Rome, 00185 Rome, Italy; floriana.cappiello@uniroma1.it (F.C.); bruno.casciaro@uniroma1.it (B.C.); 2 College of Optometry, University of Houston, Houston, TX 77204-2020, USA; sverma20@central.uh.edu (S.V.); xlin3@central.uh.edu (X.L.); iymoreno@cougarnet.uh.edu (I.Y.M.); dramcdermott@gmail.com (A.M.M.); vjcoulso@central.uh.edu (V.J.C.-T.); 3 Deen Dayal Upadhyaya College, University of Delhi, Delhi 110078, India; 4 School of Optometry and Vision Science, University of New South Wales, Sydney 2052, Australia; d.dutta@aston.ac.uk (D.D.); m.willcox@unsw.edu.au (M.W.); 5 School of Optometry, Aston University, Birmingham B4 7ET, UK

**Keywords:** antimicrobial peptides, corneal wound healing, *Pseudomonas aeruginosa*, keratitis, corneal infections, antimicrobial resistance

## Abstract

The corneal epithelium is a layer in the anterior part of eye that contributes to light refraction onto the retina and to the ocular immune defense. Although an intact corneal epithelium is an excellent barrier against microbial pathogens and injuries, corneal abrasions can lead to devastating eye infections. Among them, *Pseudomonas aeruginosa*-associated keratitis often results in severe deterioration of the corneal tissue and even blindness. Hence, the discovery of new drugs able not only to eradicate ocular infections, which are often resistant to antibiotics, but also to elicit corneal wound repair is highly demanded. Recently, we demonstrated the potent antipseudomonal activity of two peptides, Esc(1-21) and its diastereomer Esc(1-21)-1c. In this study, by means of a mouse model of *P. aeruginosa* keratitis and an in vivo corneal debridement wound, we discovered the efficacy of these peptides, particularly Esc(1-21)-1c, to cure keratitis and to promote corneal wound healing. This latter property was also supported by in vitro cell scratch and ELISA assays. Overall, the current study highlights Esc peptides as novel ophthalmic agents for treating corneal infection and injury, being able to display a dual function, antimicrobial and wound healing, rarely identified in a single peptide at the same micromolar concentration range.

## 1. Introduction

The corneal epithelium is the transparent front part of the eye, covering the iris and pupil, which serves as a clear membrane to allow light to pass into the eye, as well as providing protection to the deeper layers of the eye [1]. Unfortunately, ocular surface infections that can develop upon corneal abrasion due to accidental trauma or contact lens wear represent a serious vision threat leading to ulceration and tissue destruction if not rapidly treated within 1–2 days [2]. Furthermore, virulent bacteria colonizing the eyes such as the Gram-negative bacterium *Pseudomonas aeruginosa* can be unresponsive to traditional antibiotics [3]. *P. aeruginosa* adheres to the corneal tissue, forming biofilm communities that conventional antibiotics are not able to eradicate [4,5]. Therefore, there is a pressing need to identify novel ophthalmic agents with alternative mechanism(s) of action to treat *P. aeruginosa*. The exploitation of natural antimicrobial peptides (AMPs) has emerged as a novel strategy for treating antibiotic-resistant bacteria that cause ocular surface infections, such as keratitis.

AMPs are produced by almost all species of life as effector molecules of the innate defense system against infection [6,7]. In higher vertebrates, they also display a variety of immunomodulatory functions that confer them the most appropriate definition of host defense peptides [8]. In the amphibian anura, AMPs are synthesized by skin glands and stored within granules that are secreted in a holocrine manner upon stress or physical injury [9]. In recent years, particular interest has been focussed on the peptide Esc(1-21), [GIFSKLAGKKIKNLLISGLKG-NH_2_], corresponding to the first 20 amino acids of esculentin-1a, the AMP isolated from the skin secretion of the *Pelophylax lessonae/ridibundus* frog, formerly named *Rana esculenta* [10,11,12], with an amidated glycine at its C-terminal end [12]. It is a membrane-active peptide with the capability to perturb the target microbial membranes of both planktonic and biofilm forms of *P. aeruginosa*, likely through the formation of local breakages, thereby limiting the induction of microbial resistance [13,14].

Previous studies highlighted the efficacy of Esc(1-21) in reducing bacterial corneal infection in an appropriate mouse model of keratitis (induced by a cytotoxic strain of *P. aeruginosa,* ATCC 19660), upon eye drop instillation at 40 µM three times/day, without signs of toxicity [15]. This was the first case showing the ability of amphibian-skin-derived AMPs to successfully treat ocular surface infections. We then discovered that the diastereomer Esc(1-21)-1c, produced by changing the stereochemistry of two amino acids, Leu^14^ and Ser^17^, with the corresponding D-enantiomers, was more effective in promoting bacterial clearance in vivo (for example in a mouse model of *P. aeruginosa*-associated lung infection) [16]. In addition, Esc(1-21)-1c was more resistant than the all-L parental peptide to proteases, more efficient in preventing the development and killing of *Pseudomonas* in biofilms [17], and had a stronger antimicrobial efficacy upon conjugation to medical devices, such as hydrogel soft contact lenses [18]. However, it must be considered that a valuable treatment of damaged infected tissues implies not only the elimination of invading infectious microorganisms but also the recovery of tissue integrity and function, a process which is generally named as wound healing. Importantly, after treating the corneal infection, individuals are left with corneal epithelial wounds that are at risk of repeated infection and can lead to corneal scarring.

The aim of this study was to expand current knowledge on the ability of Esc(1-21) and its diastereomer (Esc peptides) to (i) treat keratitis elicited by an invasive bacterial strain of *Pseudomonas* and to (ii) promote corneal wound healing. Taken together, the data in this study demonstrate that Esc peptides, particularly Esc(1-21)-1c, are promising candidates for the development of novel ophthalmic agents with both antimicrobial and wound healing activity for treating ocular surface infections such as keratitis.

## 2. Materials and Methods

### 2.1. Peptides

Synthetic Esc(1-21) and Esc(1-21)-1c were purchased from Biomatik (Wilmington, NC, USA). Each peptide was assembled by stepwise solid-phase synthesis carrying out a standard F-moc protocol and subsequently purified via reverse-phase high-performance liquid chromatography (RP-HPLC) to a purity of 98%. Mass spectrometry was employed to check the molecular mass [19].

### 2.2. Bacterial Strains and Antimicrobial Activity

The reference invasive strain of *P. aeruginosa*, 6294, previously reported to induce microbial keratitis in mice, was used [20]. Susceptibility testing was performed by adapting the microbroth dilution method outlined by the Clinical and Laboratory Standards Institute using sterile 96-well plates. Aliquots (50 μL) of bacteria in the mid-log phase at a concentration of 2 × 10^6^ colony-forming units (CFU)/mL in Muller–Hinton (MH) broth were added to 50 μL of MH broth containing the peptide in serial two-fold dilutions. The range of peptide concentrations used was 0.125–64 μM, each in triplicate. Inhibition of microbial growth was determined by measuring the absorbance at 590 nm, after incubation for 18 h at 37 °C with a microplate reader (Infinite M200; Tecan, Salzburg, Austria). The antimicrobial activities were expressed as minimal inhibitory concentration (MIC) values, the concentration of peptide at which 100% inhibition of microbial growth is observed.

### 2.3. In Vivo Infection

*P. aeruginosa* strain 6294 was grown overnight at 37 °C in Tryptone Soya broth (TSB; Oxoid, Basingstoke, UK) and then washed with phosphate-buffered saline (PBS). The *P. aeruginosa* pellet was resuspended in PBS to an optical density (OD) at 600 nm of 0.1 (1.0 × 10^8^ CFU/mL) [21]. The bacterial suspension was serially diluted to 1.0 × 10^6^ CFU/mL for the animal model experiment. A *P. aeruginosa* suspension was freshly prepared for each experiment, where only PBS was used as the vehicle control.

This study received approval from the Institutional Animal Ethics Committee (ACEC number 17/98A) at the University of New South Wales, Australia. The mice were treated according to the statement for the Use of Animals in Ophthalmic and Vision Research by Association for Research in Vision and Ophthalmology (ARVO). C57BL/6 female mice aged between 7–10 weeks were used in this study. All mice were acclimatised for more than 7 days in the research facility, and the baseline ocular examinations were conducted under anaesthesia following intraperitoneal injections of ketamine (100 μg/mL) and xylazine (1 μg/mL). After anaesthesia, three vertical scratches were made on the corneal surface using a 25G needle and 10 µL of 10^6^ CFU/mL *P. aeruginosa* inoculum was instilled on the cornea. All procedures were done in the left eye and the right eye was left untreated. All infected animals were given the analgesia buprenorphine (Provet, Sydney, Australia; 0.1 mg/kg of body weight) subcutaneously twice a day until the end of the study. The animals were divided into three groups, namely the (1) control group with PBS (vehicle control), (2) Esc(1-21) treatment group, and (3) Esc(1-21)-1c treatment group, and three animals were allocated for each group. The fourth was processed as the control corneal scratch group, where only one animal was used per experiment and *P. aeruginosa* inoculum was not applied. After five hours, all infected animals except the process control developed microbial keratitis and treatment was initiated. One drop of 40 µM of Esc(1-21) and Esc(1-21)-1c dissolved in PBS or only PBS was applied in the left eye three times a day for the next three days. All animals were restrained for a minute to prevent the loss of the eye drop. After this the eyes were examined and photographed daily using a video slit-lamp (Zeiss SL 115 Classic, NSW, Australia). The grading of the ocular inflammation was done using a clinical score range of 0–4 (0 = clear cornea; 4 = corneal perforation), which has been reported earlier [15]. Mice with an infection and inflammation grade of 4 or more were immediately euthanised. All animals were examined three times a day, all were euthanised after the end of the experiment, and the whole experiment was repeated twice.

### 2.4. In Vivo Corneal Debridement Wound

Seven- to ten-week-old C57BL/6 mice were provided with Rimadyl (Carprofen, 2 mg/tablet, Rodent MD’s, Bio-Serv, Flemington, NJ, USA) 24 h prior to the debridement wound. Before wounding, the mice were anesthetized by intraperitoneal injections of ketamine and xylazine (Akorn Animal Health, Gurnee, IL, USA) with doses of 80 mg/kg and 10 mg/kg, respectively. The eyes were briefly rinsed with sterile PBS and 0.5% proparacaine (Bausch and Lomb, Bridgewater, NJ, USA) was administered to the eye after removing the excess of PBS using an absorbent sponge. Furthermore, 1.5 mm of trephine (Robbins Instruments, Chatham, NJ, USA) was used to demarcate the area to be debrided. The debridement wound was made in the central cornea of both eyes and the epithelium was carefully removed to minimize damage to the basement membrane using an Algerbrush II instrument (Alger Company, Inc., Lago Vista, TX, USA) with a 0.5 mm rotating burr. The injured ocular surface was imaged using a ring light (Schott, VisiLED M1100, New York, NY, USA) and the wounded area was imaged using fluorescein dye (GloStrips; Amcon Laboratories Inc., St. Louis, MO, USA) under a ZEISS SteREO Discovery.V12 Modular Stereo Microscope with the GFP filter (Carl Zeiss Microscopy LLC, Oberkochen, Germany). The right eye was treated with the peptides at concentrations of 10 µM, 40 µM, and 100 µM, while the left eye was provided with PBS to serve as the control. The peptides were maintained on the ocular surface for the first 2 h, and thereafter the mouse was allowed to recover from anaesthesia and returned to its cage. At 12 h and 24 h, the mice were anesthetized, the injured area was imaged again using fluorescein and a ring light, and an eye drop was maintained on the ocular surface for an additional 30 min. The injured area was quantified using ImageJ software.

### 2.5. Human Corneal Epithelial Cell Culture Conditions

The human-telomerase-immortalized corneal epithelial cell line (hTCEpi) was kindly provided by Dr. James Jester, UC Irvine [22]. The hTCEpi was maintained in serum-free keratinocyte culture medium (DermaLife^®^ Basal media kit; Lifeline Cell Technology, Frederick, MD, USA) containing supplements provided in the DermaLife Basal media kit with final concentrations of 6 mM of L-Glutamine, 1.0 μM of epinephrine, 5 μg/mL of recombinant human TGF-α, 100 ng/mL of hydrocortisone hemisuccinate, 5 μg/mL of rh-insulin, 5 μg/mL of apo-transferrin, 30 mg/mL of gentamicin, 0.4% bovine pituitary extra, and 15 μg/mL of amphotericin B at 37 °C in 5% CO_2_. The cells were sub-cultured when reaching 70–90% confluence with 0.25% trypsin-EDTA (Gibco, Thermo Fisher Scientific, Waltham, MA, USA).

### 2.6. In Vitro Cell Scratch Assay

The hTCEpi cells were seeded in 24-well plates at a density of 92,000 cells/well and left at 37 °C and 5% CO_2_ until reaching confluence. For the scratch assay, the cells were mechanically removed from the confluent monolayer by dragging a 200 μL pipette tip linearly down the middle of the well using a guide as a reference. Two sequential PBS washes were used to remove any loose cells or debris, and fresh medium was placed into the well containing or not containing the peptides at different concentrations. The peptides were tested at concentrations of 2.5 μM, 20 μM, and 100 μM and a medium containing PBS was used as the control. The microplate was placed into a 24-well plate with a heated insert in an PM 2000 RBT incubator (Zeiss) at 5% CO_2_ and 37 °C, and the scratched edges were imaged after 6, 12, 24, and 30 h under a ZEISS LSM 800 confocal microscope (Zeiss) using the time-elapse module. The wounded area was quantified using an Image J script established for in vitro wound healing assays.

### 2.7. Proliferation Assay

The effects of the peptides at concentrations of 2.5 μM, 20 μM, and 100 μM on the proliferation of the hTCEpi were assessed using a BrdU (5-bromo-2′-deoxyuridine) Cell Proliferation Assay Kit (EMD Millipore, MA, USA), according to the manufacturer’s instructions. In short, hTCEpi cells were seeded in a 96-well plate at a density of 3600 cells per well. After 24 h, BrdU was added to the culture media and the plates were incubated for an additional 6 h, after which the cells were fixed using the provided fixing solution. The incorporated BrdU was detected using the provided anti-BrdU monoclonal antibody. Absorbance was measured as OD at 450 nm using a microplate reader (FLUOstar Omega; BMG Labtech, Ortenberg, Germany). The medium only was used as the blank, and cells in the media were used as the control.

### 2.8. Cell Viability Assay

The viability of hTCEpi cells was assessed using the Cell Counting Kit 8 (CCK-8) (APExBIO, Houston, TX, USA) according to the manufacturer’s instructions. Briefly, aliquots of cell suspensions were added to the wells of a flat-bottom 96-well plate (5000 cells/well). The plate was pre-incubated for 24 h in a humidified incubator at 37 °C and 5% CO_2_. Afterwards, 10 μL volumes of various concentrations (10, 40, and 100 μM) of Esc(1-21)-1c were added to the culture medium in the plate. The culture medium only was taken as the blank control. The plate was incubated for 24 h. Then, a 10 μL solution of CCK-8 was added to each well, avoiding air bubbles. The plate was incubated for 3 h and the absorbance measured at 450 nm with a microplate reader. Cell viability was expressed as a percentage with respect to the untreated control cells.

### 2.9. Enzyme-Linked Immunosorbent Assays (ELISA)

The hTCEpi cells were seeded in 96-well plates at a density of 15,000 cells/well. After overnight incubation at 37 °C and 5% CO_2_, the cells were treated with different concentrations of Esc peptides for 24 h; cells in the media were used as controls. At the end of the treatment, the supernatants were centrifuged for 20 min at 1000× *g*, then collected and stored at −20 °C for later use. ELISA assays were performed according to the manufacturer’s protocol to detect the following cytokines or growth factors in the cell supernatants: interleukins (IL)-6 and -1β (Cloud-Clone Corp., Houston, TX, USA), transforming (TGF)-β1 (LEGEND MAX, Biolegend, San Diego, CA, USA), and platelet-derived (PDGF)-BB growth factors (Proteintech, Wuhan, China).

### 2.10. Statistical Analyses

The quantitative data collected from independent experiments are expressed as the means ± standard deviation (SD) or standard errors of the means (SEM). A statistical analysis was performed using a one-way or two-way analysis of variance (ANOVA) with PRISM 8.0.1 software (GraphPad, San Diego, CA, USA). Differences were statistically significant for *p* values < 0.05. The levels of statistical significance are indicated in the legends to the figures.

## 3. Results

### 3.1. Antipseudomonal Activity of Esc Peptides In Vitro and in a Mouse Model of Keratitis

The susceptibility of *P. aeruginosa* 6294 to increasing concentrations of Esc peptides was studied by the microdilution broth assay to determine the MIC. From the observation of samples, clear or non-turbid wells corresponding to the MIC were found at 5 μM and 20 μM for Esc(1-21) and Esc(1-21)-1c, respectively.

**Figure 1:**
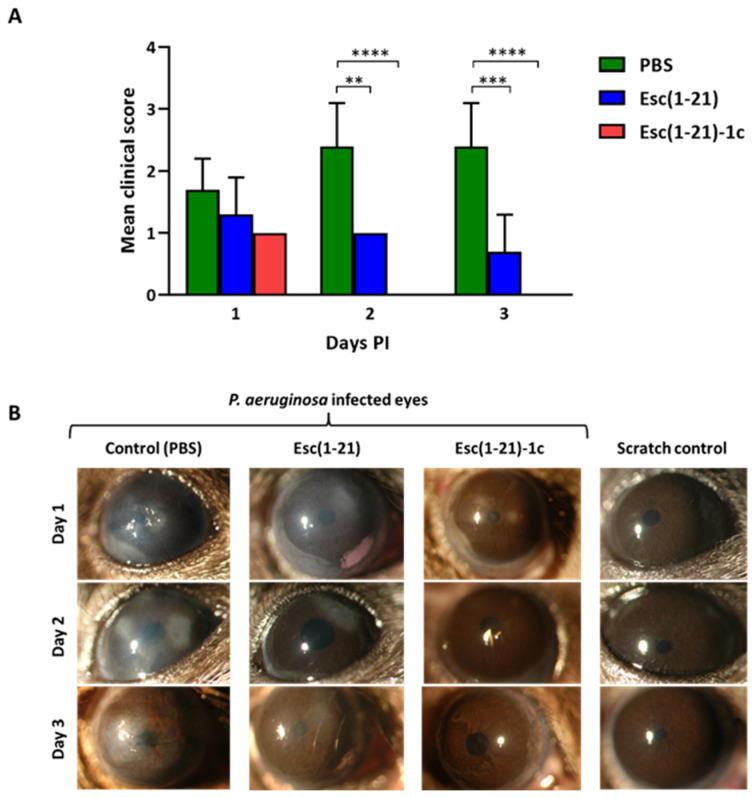
Effects of Esc peptides on *P. aeruginosa* keratitis in C57BL/6 mice following day 1, day 2, and day 3. (**A**) Clinical scores indicated that eyes treated with Esc(1-21) and Esc(1-21)-1c had significantly reduced severity compared to PBS-treated (control) animals. Data represent the means ± SD; the levels of statistical significance among groups were determined by a two-way ANOVA and are indicated as follows: ** *p* < 0.01; *** *p* < 0.001; **** *p* < 0.0001. (**B**) Representative photographs indicate that corneal haze and infiltration in control group was higher on day 1, day 2, and day 3 compared to treatment groups. By day 3, almost all animals recovered from keratitis; however, the eyes in the control group showed higher corneal haze rates, a result of severe keratitis.

Based on previous efficacy data for Esc(1-21) against *P. aeruginosa*-induced keratitis in a mouse model [15], the corneal surface of the mice was scratched, infected with *P. aeruginosa* ATCC 6294, and then treated or not with the peptides; clinical progression of the corneal infection was examined by slit lamp bio-microscopy. All *P. aeruginosa*-infected animals developed microbial keratitis within five hours of inoculation and none developed perforation by the end of the three-day study. The peptide-treated mice showed significantly better clinical outcomes with reduced corneal opacity and infiltration at every stage of the study (Figure 1). The control infected mice had clinical scores of 1.7 ± 0.5, 2.4 ± 0.7, and 2.4 ± 0.7 on days 1, 2, and 3 respectively, indicating a disease with rapid progression. In comparison, the peptide treatment significantly reduced the level of infection, as shown by the slight opacity partially covering the ocular surface 2 days post-infection (PI) for Esc(1-21)-treated animals (the clinical scores were 1.3 ± 0.6, 1.0 ± 0.0, and 0.7 ± 0.6 at days 1, 2, and 3 PI, respectively), whereas the cornea was clear and transparent, similar to non-infected control eyes, for the eyes treated with the Esc(1-21)-1c diastereomer (the clinical score were 1.0 ± 0.0, 0.0 ± 0.0, and 0.0 ± 0.0 at days 1, 2, and 3 PI, respectively), which led to recovery from infection.

### 3.2. In Vivo Corneal Wound Healing

**Figure 2:**
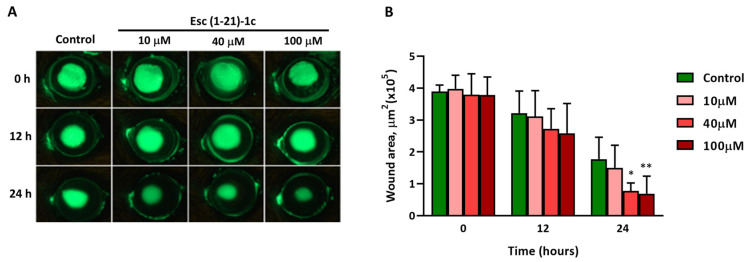
Assessment of effects of Esc(1-21)-1c peptide on corneal epithelial wound healing in mice using fluorescein staining: (**A**) representative images of fluorescein stained PBS-treated (control) and peptide-treated (Esc(1-21)-1c at 10 µM, 40 µM, and 100 µM concentrations) corneas after 0 h, 12 h, and 24 h of epithelial debridement; (**B**) a quantitative analysis of the time course of the corneal epithelial wound area (µm^2^) at specified time points after debridement. Data represent means ± SD and asterisks denote significant differences vs. control: * *p* < 0.05 and ** *p* < 0.01.

**Figure 3:**
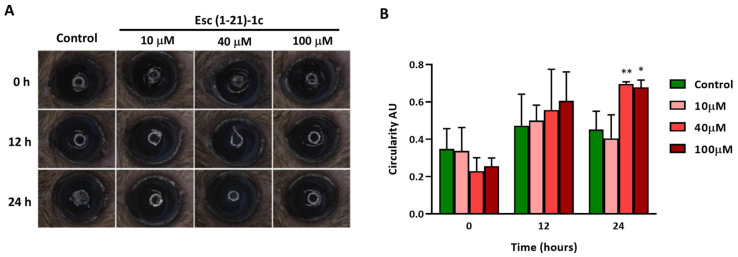
The effects of the Esc(1-21)-1c peptide on corneal wound healing by evaluating the smoothness of the cornea using a white ring light projected onto the ocular surface: (**A**) representative images of PBS-treated (control) and peptide-treated (Esc(1-21)-1c at 10 µM, 40 µM, and 100 µM concentrations) corneas after 0 h, 12 h, and 24 h of epithelial debridement, using a white ring light under a stereomicroscope; (**B**) the circularity of the projected ring light was quantified from an image captured for each mouse to quantify the corneal smoothness using Image J 1.52p (National Institutes of Health) software and data presented as a time course of the circularity index. Data represent means ± SD and asterisks denote significant differences vs. control: * *p* < 0.05 and ** *p* < 0.01.

The most active peptide Esc(1-21)-1c was then evaluated for its ability to enhance corneal epithelial wound healing in vivo (Figure 2A). At the two timepoints analyzed, the scratched area was significantly smaller in the peptide-treated eyes than in the vehicle (PBS)-treated ones (control). By 24 h, eyes treated with 40 µM and 100 µM showed a significant reduction in the wounded area compared to the control animals group, highlighting the effectiveness of Esc(1-21)-1c in promoting corneal wound healing (Figure 2B). Additionally, an intact corneal epithelial layer formed a smooth surface over the cornea, which was assessed by analyzing the circularity of a projected ring light onto the ocular surface (Figure 3). At 24 h after injury, corneas treated with the peptides at the 40 and 100 µM concentrations presented a significantly smoother cornea when compared to PBS, indicating less epithelial damage.

### 3.3. In Vitro Wound Healing on Human Corneal Epithelial Cells

**Figure 4:**
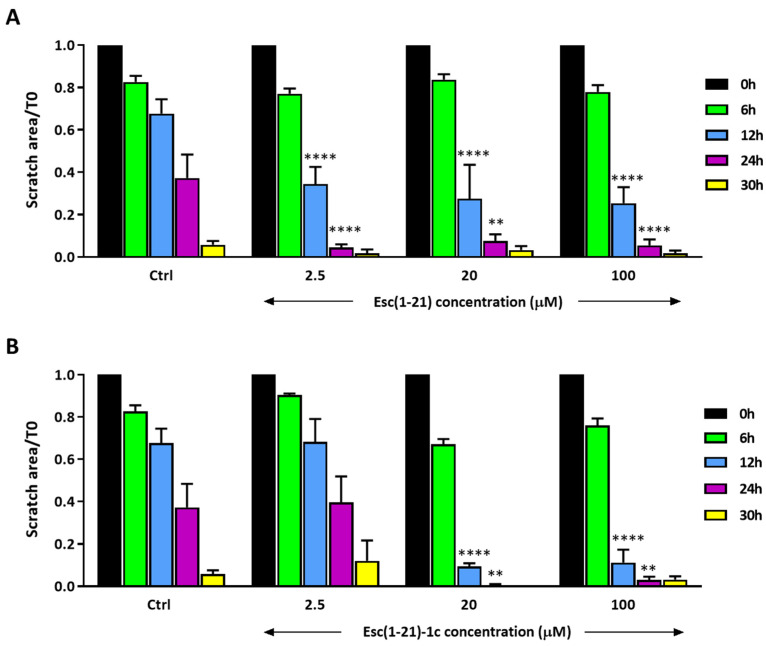
Effects of Esc(1-21) (**A**) and its diastereomer Esc(1-21)-1c (**B**) on the closure of a pseudo-wound field produced in a monolayer of hTCEpi cells by the scratch assay. Samples were photographed at the time of insert removal (T0) and checked for cell migration after 6, 12, 24, and 30 h. The percentage of the cell-covered area at each time point is indicated on the *y*-axis. Control (Ctrl) represents cells not treated with the peptide. All data are the means of three independent experiments ± SEM. The level of statistical significance among groups was determined by a two-way ANOVA and is indicated as follows: ** *p* < 0.01 and **** *p* < 0.0001.

To further study how Esc(1-21)-1c affects different aspects of corneal wound healing, its efficacy in promoting human corneal epithelial cell migration was verified using an in vitro scratch assay. For such, a scratch was produced in a confluent monolayer of hTCEpi cells, and thereafter the cells were treated or not with the Esc peptides and imaged after 6, 12, 24, and 30 h (Figure 4). Starting from 12 h of treatment, the Esc peptides facilitated closure of the gap area in a dose- and time-dependent manner, with Esc(1-21)-1c application resulting in significantly smaller scratch areas at 20 μM.

**Figure 5:**
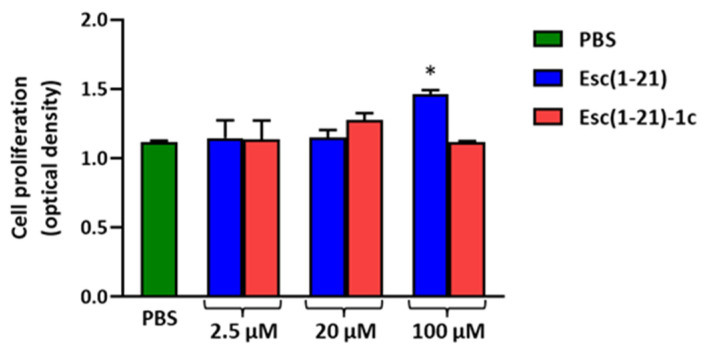
Evaluation of cell proliferation after 24 h treatment with Esc peptides at different concentrations. All data are the means of three independent experiments ± SEM. Significance level between peptide-treated samples and PBS treated cells, used as control, is defined as follows: * *p* < 0.05.

Given that the Esc peptides significantly promoted corneal wound healing, their effects on corneal epithelial cell proliferation were then investigated using the BrdU assay. No significant cell proliferation was detected (Figure 5), except for Esc(1-21) at 100 μM. Note, however, that even if Esc(1-21) is better able to stimulate the in vitro proliferation of corneal epithelial cells at 100 μM, compared to Esc(1-21)-1c, it is not as efficient in stimulating in vitro corneal wound healing (Figure 4). This highlights that the wound healing activity induced by Esc peptides is mainly due to cell migration (rather than to cell proliferation), according to previous studies performed on human bronchial epithelial cells and keratinocytes [23,24]. The most efficient peptide Esc(1-21)-1c was also assessed for its in vitro cytotoxicity on the hTCEpi. As expected, it did not provoke any harmful effect (100% cell viability over a concentration range of 10 to 100 μM within 24 h of peptide treatment; Figure S1). 

### 3.4. Cytokine Production

To explore whether Esc peptides induced the expression of cytokines, which play a relevant role as regulators of corneal epithelial wound healing, the level of cytokines secreted by hTCEpi cells upon exposure to the peptides was also monitored. To this end, supernatants collected from hTCEpi cells treated or not with different concentrations of each peptide for 24 h were analyzed to quantify the level of interleukins (IL)-6 and -1β, transforming (TGF)-β1, and platelet-derived (PDGF)-BB growth factors by ELISA.

**Figure 6:**
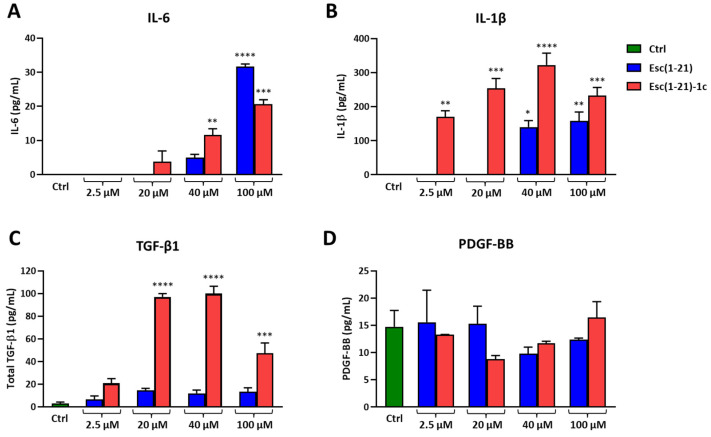
Effect of Esc(1-21) and Esc(1-21)-1c on cytokine and growth factor production by hTCEpi cells after 24 h of treatment. An ELISA was performed to measure the IL-6 (**A**), IL-1β (**B**), total TGF-β1 (**C**), and PDGF-BB (**D**) levels in supernatants. Cells treated with media served as the control (Ctrl). Results are expressed as the means ± SEM of three independent experiments, each performed in duplicate. The levels of statistical significance between the Ctrl and samples treated with each peptide was determined by a one-way ANOVA and are indicated as follows: * *p* < 0.05, ** *p* < 0.01, *** *p* < 0.001, and **** *p* < 0.0001.

As reported in Figure 6A, both peptides stimulated the secretion of IL-6 in a dose-dependent manner within 24 h; the effect was more pronounced for Esc(1-21) at the highest concentration tested (100 µM), while in the case of Esc(1-21)-1c, the IL-6 production significantly increased either at 40 µM or 100 µM.

The IL-1β secretion increased in cell supernatants after Esc(1-21) treatments at 40 µM and 100 µM (Figure 6B). However, the greatest effect was obtained with Esc(1-21)-1c, which was able to stimulate IL-1β release at all concentrations tested. At 40 µM, Esc(1-21)-1c induced more than two-fold production of IL-1β compared to the all-L peptide (Figure 6B). No secretion of either IL-6 or IL-1β was found for the control cells, in line with what reported in the literature for untreated hTCEpi cells [25,26]. In addition, a significant increase in total TGF-β1 was found after Esc(1-21)-1c treatment with respect to the control, at the concentration range between 20 µM and 100 µM (Figure 6C). In comparison, no statistically significant difference was found between the control and samples treated with both peptides, at all concentrations, when the supernatants were analyzed for secreted PDGF-BB (Figure 6D).

## 4. Discussion

Bacterial keratitis is an ophthalmic emergency that can progress rapidly to visual impairment, with the highest prevalence in contact lens wearers in developing and industrialized countries [27,28]. The most common cause of contact-lens-related keratitis is *P. aeruginosa,* and its two types, invasive or cytotoxic, cause approximately 50% each of the cases [29,30]. It is commonly associated with risk factors that disturb the corneal epithelial integrity [31] and is the principal cause of blindness globally, resulting in unilateral vision loss in an estimated 2 million people per year according to the World Health Organization reports [32]. Therefore, the enhancement of corneal epithelial wound healing is highly desirable to prevent infection and loss of vision after injury. A topically applied treatment able to stimulate corneal epithelial cell migration and to reduce corneal infection would be very helpful for the management of corneal wounds and keratitis.

Corneal wound healing is a complex series of events involving several stages, including cell migration, proliferation, cell matrix adhesion, and tissue remodelling [33]. AMPs such as LL-37 and defensins are present at the ocular surface and have been shown to have a key role in the modulation of essential processes in corneal epithelial wound healing [33]. AMPs can stimulate wound tissue regeneration in different ways. For example, LL-37 improved the granulation tissue formation and re-epithelialization in a mouse excisional wound model [34] and was found to promote the healing of deep tissue injury, especially when incorporated into chitosan hydrogel [35]. The peptide epinecidin-1 healed wounds by increasing the synthesis of the extracellular matrix [36,37].

Esc peptides were previously reported to have an in vitro wound healing activity on keratinocytes and bronchial epithelial cells [23,24]. There was epidermal growth factor receptor (EGFR)-mediated activation as well as the involvement of MMP-9 metalloprotease in the Esc-peptide-mediated migration of bronchial epithelial cells [38]. Recently, a paper highlighted the in vivo wound healing activity of the N-terminal acetylated form of Esc(1-21) in a full-thickness excision mouse model [39]. The peptide was able to accelerate wound closure when locally administered at 100 μM, once a day.

We hereby show that the instillation of eye drops containing the diastereomer Esc(1-21)-1c at 40 µM is highly efficient at fighting *P. aeruginosa*-induced keratitis, leading to full recovery from corneal infection within 2 days of treatment; a stronger effect than that elicited by the all-L parental Esc(1-21), despite the higher MIC in vitro (20 μM vs. 5 μM of Esc(1-21)). This is likely due to the higher biostability of the D-amino-acid-containing diastereomer and prolonged residence time, which make it more active than the all-L isoform [16,17]. In addition, these findings correlate well with the higher in vivo efficacy of Esc(1-21)-1c in reducing *P. aeruginosa’s* burden in a mouse model of acute lung infection upon intratracheal administration, either in its soluble free form or encapsulated into poly(lactide-co-glycolide) (PLGA) nanoparticles, compared to the all-L peptide [40]. Remarkably, new nanotechnology-based antimicrobials have been designed and developed as promising tools to tackle bacterial infections that are difficult to treat, including those provoked by biofilms of multi-drug-resistant strains [41,42,43]. In particular, Esc-peptide-loaded PLGA nanoparticles were produced and optimized as inhaled formulations for the pulmonary delivery of AMPs upon administration in the conductive airways and to assist their transport across bio-barriers imposed by the lungs [40]. Further studies are certainly needed to identify proper formulations suitable for the ocular delivery of Esc peptides with the aim of facilitating their diffusion through the tear film and to extend and ameliorate their therapeutic efficacy.

Furthermore, in this work, Esc(1-21)-1c was found to promote corneal wound healing in a dose-dependent manner, primarily through increasing the migration of corneal epithelial cells (rather than eliciting proliferation) and leading to an accelerated rate of wound closure. In the context of wound re-epithelialization, several cytokines (i.e., IL-6, IL-1β) are released by epithelial cells to initiate a cascade of intercellular responses helpful in the coordination of the corneal healing, including cellular and tissue contraction as well as extracellular matrix deposition and the production of appropriate growth factors [44,45,46,47,48]. The current experiments have indicated statistically significant secretion of IL-6 and IL-1β from hTCEpi cells at concentrations able to stimulate in vitro corneal wound healing. Interestingly, the treatment of corneal epithelial cells with Esc(1-21)-1c, which was the most efficient peptide in stimulating the in vitro re-epithelialization of the corneal epithelial cell monolayer, strongly increased the level of secreted TGF-β1, which is known to play a crucial role in the promotion of corneal epithelial cell migration [49,50,51,52,53]. In comparison, no secretion of PDGF-BB was obtained from hTCEpi cells upon exposure to Esc peptides, in line with that previously observed in mesenchymal cells of the corneal stroma during wound re-epithelialization [54].

Overall, this work demonstrates the in vivo efficacy of Esc peptides, particularly the diastereomer Esc(1-21)-1c, in treating *P. aeruginosa* keratitis, along with their in vitro and in vivo corneal wound healing activity. It is worthwhile noting that so far only a few studies have reported on the AMPs displaying a dual function, e.g., antimicrobial and wound healing activity at the ocular surface. For example, histatin 5 was found to speed up (i) cell migration in a standardized scratch assay of human corneal epithelial cells and (ii) wound closure in a murine corneal epithelial injury model when applied dropwise at 80 µM three times per day. Under such conditions, it reduced the percentages of the wound area to 40 and 20% after 18 and 24 h, respectively [55]. In comparison, Esc(1-21)-1c provoked a similar outcome (20% wound area after 24 h treatment) when instilled on the ocular surface at 20 μM only twice per day. However, histatin does not have any activity against *P. aeruginosa*. A hybrid peptide of cecropin A and melittin (derived from the venom of the European honey bee) exhibited activity in a rabbit model of *P. aeruginosa*-induced keratitis [56], although no indication on the wound healing ability was disclosed. Furthermore, a derivative of the peptide CAP37 was recently shown to be effective in clearing *P. aeruginosa* corneal infection as well as in accelerating corneal wound healing in vivo, although the concentrations used for the antibacterial activity were significantly higher (in the range of mM) compared to those displaying wound healing activity (in the µM range) [57]. However, hybrid derivatives of cathelicidin and human beta defensin-2 manifested an in vivo efficacy for the treatment of keratitis associated with the Gram-positive bacterium *Staphylococcus aureus*—together with the ability to stimulate corneal wound healing at relatively high concentrations (above 100 µM) [58].

## 5. Conclusions

Remarkably, the current study confirmed the efficacy of Esc peptides in treating ocular surface *P. aeruginosa*-associated infections, even when an invasive bacterial strain was used. Moreover, we have discovered how a derivative of naturally occurring AMP-containing non-coded D-amino acids represents a valid alternative for the development of new ophthalmic agents, being able not only to eradicate ocular surface *Pseudomonas* infections but also to elicit corneal wound repair by promoting the secretion of cytokines and growth factors associated with corneal wound healing at the same micromolar concentration range—two remarkable features for the successful treatment of eye dysfunctions, such as keratitis, characterized by both corneal infection and injury.

## Data Availability

Data is contained within the article and Supplementary Material.

## Supplementary Materials

The following supporting information can be downloaded at: https://www.mdpi.com/article/10.3390/biom13071028/s1. Figure S1. Effect of Esc(1-21)-1c on the viability of hTCEpi cells.
