# Supplementary material for: Novel Peptides with Dual Properties for Treating Pseudomonas aeruginosa Keratitis: Antibacterial and Corneal Wound Healing
Source: Biomolecules. 2023 Jun 23;13(7):1028. doi: 10.3390/biom13071028 (PMC10377436; doi:10.3390/biom13071028)
Supplement: Supplementary file 1 [file biomolecules-13-01028-s001.zip › biomolecules-2437363-supplementary.pdf]

biomolecules

MDPI

Type of the Paper (Article)

Novel peptides with dual properties for treating *Pseudomonas aeruginosa* keratitis: anti-bacterial and corneal wound healing

Floriana Cappiello [1†], Sudhir Verma [2,3†], Xiao Lin[2], Isabel Y. Moreno[2], Bruno Casciaro[1], Debarun Dutta[4,5], Alison M. McDermott[2], Mark Willcox[4], Vivien J Coulson-Thomas[2] and Maria Luisa Mangoni [1,*]

[1] Laboratory affiliated to Pasteur Italia-Fondazione Cenci Bolognetti, Department of Biochemical Sciences, Sapienza University of Rome, Italy; floriana.cappiello@uniroma1.it (F.C.), bruno.casciaro@uniroma1.it (B.C.), marialuisa.mangoni@uniroma1.it (M.L.M.)

[2] College of Optometry, University of Houston, Houston, Texas, USA; sverma20@central.uh.edu (S.V.), xlin3@central.uh.edu (X.L.), iymoreno@CougarNet.UH.EDU (I.Y.M.), dramcdermott@gmail.com (A.M.M); vjcoulso@central.uh.edu (V.J.C.-T.)

[3] Deen Dayal Upadhyaya College, University of Delhi, Delhi, India; sverma20@central.uh.edu (S.V.)

[4] School of Optometry and Vision Science, University of New South Wales, Sydney, Australia; d.dutta@aston.ac.uk (D.D.), m.willcox@unsw.edu.au (M.W.)

[5] School of Optometry, Aston University, Birmingham, UK; d.dutta@aston.ac.uk (D.D.)

† These authors contributed equally to this work

* Correspondence: marialuisa.mangoni@uniroma1.it (M.L.M); Tel.: +39 06 49910838 Laboratory affiliated to Pasteur Italia-Fondazione Cenci Bolognetti, Department of Biochemical Sciences, Sapienza University of Rome, Italy-00185

Citation: To be added by editorial staff during production.

Academic Editor: Firstname Lastname

Received: date
Revised: date
Accepted: date
Published: date

Copyright: © 2023 by the authors. Submitted for possible open access publication under the terms and conditions of the Creative Commons Attribution (CC BY) license (https://creativecommons.org/licenses/by/4.0/).

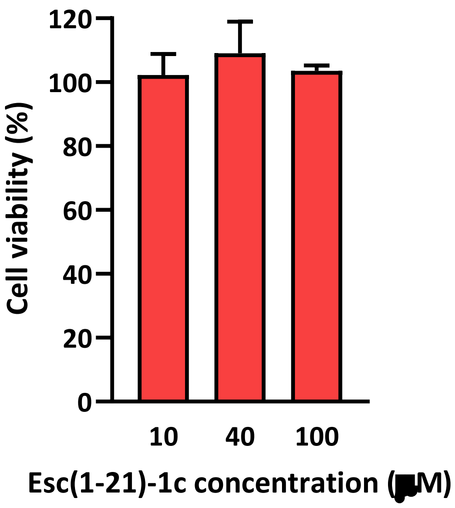

Figure S1

Effect of Esc(1-21)-1c on the viability of hTCEpi cells. One hundred µL of media containing 5,000 cells were plated in each well of a 96-well plate. After 24 h incubation at 37 °C in a 5% CO2 atmosphere, 10 µL of peptide solution were added into the culture medium of each well to obtain the final concentration of 10, 40 or 100 µM. Cells incubated with media only were used as control. Afterwards, 10 µL of CCK-8 solution were added to each well. The plate was incubated for 3 h. Finally, the absorbance was measured at 450 nm using a microplate reader. Cell viability is expressed as a percentage with respect to the control. All data are the means of three replicates ± SEM.
